# Detection of Geographic Atrophy Guided by Optical Coherence Tomography Sub-RPE Illumination Analysis in Patients With Intermediate Age-Related Macular Degeneration

**DOI:** 10.1177/24741264241305107

**Published:** 2024-12-30

**Authors:** Andrei Szigiato, Christopher M. Maatouk, Alexander E. Azar, Peter Alsaloum, Katherine E. Talcott, Rishi P. Singh, Aleksandra V. Rachitskaya

**Affiliations:** 1 Cole Eye Institute, Cleveland Clinic, Cleveland, OH, USA; 2 Case Western Reserve University School of Medicine, Cleveland, OH, USA; 3 Cleveland Clinic Martin Hospitals, Cleveland Clinic Florida, Stuart, FL, USA

**Keywords:** intermediate age-related macular degeneration, AMD, geographic atrophy, GA, atrophy, iRORA, cRORA, OCT, subfield illumination area, sub-RPE illumination

## Abstract

**Purpose:** To evaluate the prevalence of geographic atrophy (GA) lesions in patients with a diagnosis of intermediate age-related macular degeneration (iAMD). **Methods:** A retrospective cross-sectional study was performed of patients with an International Classification of Diseases, 10th Revision, diagnosis of iAMD. The primary outcome was the percentage of eyes diagnosed with iAMD with an undocumented GA lesion identified on imaging. Multiple logistic regression was used to assess risk factors for atrophic lesions in patients with iAMD. **Results:** The study included 690 eyes of 428 patients with a diagnosis of iAMD. The mean age was 79.4 ± 8.4 years, and 66.3% of patients were women. Forty-nine eyes (7.1%) were graded as having GA lesions, and 34% of these eyes had foveal involvement. The mean visual acuity (VA) was better in patients without GA lesions than in patients with GA lesions (72.9 ± 12.9 letters vs 66.4 ± 13.8 letters; *P =* .001). No systemic comorbidity was associated with an increased risk for GA lesions in this cohort. **Conclusions:** A notable proportion of eyes diagnosed with iAMD by eye care providers had underlying GA lesions in this real-world cohort. The use of optical coherence tomography as an adjunctive tool helped increase the detection of early GA in these patients.

## Introduction

Geographic atrophy (GA) is an advanced form of age-related macular degeneration (AMD) and is one of the leading causes of blindness in the developed world, with an estimated 1 million people in the United States affected.^1^ The natural history of GA involves the progressive loss of photoreceptors, retinal pigment epithelium (RPE) cells, and the choriocapillaris, manifesting as well-demarcated round or oval areas of atrophy on clinical examination.^2^ An insidious disorder, GA features atrophic lesions in the early stages and typically involves the parafoveal retina. These lesions can later grow into the fovea, leading to the loss of central vision and ultimately impairing daily activities such as reading, driving, and recognizing faces.^3^

In February 2023, the first treatment for GA was approved by the US Food and Drug Administration (FDA).^4^ Pegcetacoplan, or APL-2, is a synthetic cyclic peptide inhibitor of complement C3, a key mediator of the complement system that has been found to be elevated in patients with AMD.^5,6^ Previous cohort studies have shown a large variation in the rate of progression of extrafoveal GA to foveal GA and thus increased visual disability, with an average of approximately 5.6 years after presentation.^7^ Phase 3 data showed that intravitreal (IVT) injection with pegcetacoplan reduced the rate of GA lesion growth. In the DERBY clinical trial, monthly treatment led to a reduction in lesion growth by 36% after 18 to 24 months; a 29% reduction in lesion growth was found in patients treated every other month (*P* < .001).^8^ In the OAKS clinical trial, monthly treatment resulted in a 24% reduction in lesion growth after 18 to 24 months and a 25% reduction in lesion growth in patients treated every other month (*P* < .001).^8^ Avacincaptad pegol, an inhibitor of complement C5, has also been shown to reduce the growth of atrophic lesions by 14% compared with a sham when administered monthly via IVT injection after 12 months.^9^

Although pegcetacoplan and other treatments slow the growth of atrophy, the existing atrophic lesions are not affected. Timely treatment of GA has potential benefits, emphasizing the need to identify patients with early signs of the condition who are at risk for vision loss in the coming years.

Multimodal imaging can greatly aid in the identification of atrophic lesions. Fundus autofluorescence (FAF) is the gold standard to assess for the presence and progression of atrophy and is being used in most GA clinical trials. Spectral-domain optical coherence tomography (SD-OCT) is an essential, nearly ubiquitous tool that captures a compilation of B-scans and generates a 3-dimensional dataset that can be used to construct an image of the macula. The resulting en face OCT fundus image provides a valuable tool for visualizing GA, which appears as a bright area as a result of enhanced light penetration into the choroid where macular atrophy has occurred. The increased OCT signal associated with GA arises from the absence of the RPE and choriocapillaris layers, which normally induce light scattering and limit light transmission into the deeper choroidal layers. The brightness observed on the OCT fundus image is a culmination of this heightened light penetration relative to the surrounding tissue, which still possesses intact RPE and choriocapillaris layers.^10,11^

Although FAF is the traditional imaging modality for GA lesion measurement, a more recent approach to GA imaging is the use of the sub-RPE illumination analysis, or the sub-RPE slab, using OCT.^12^ An en face image is created using light penetrating beneath the RPE and into the choroid. The analysis draws automatic boundaries in these areas of hypertransmission that correlate well with manual reshading in large GA lesions (ICC, 0.998).^12^ Previous studies comparing SD-OCT and FAF showed a similar measurement of a GA lesion area with manual correction of segmentation errors (*r* = 0.98), suggesting that OCT is also an effective method of characterizing GA lesions.^13–15^

The purpose of this study was to use OCT imaging analysis to evaluate the prevalence of GA lesions in patients with an International Classification of Diseases, 10th Revision (ICD-10), diagnosis of intermediate AMD (iAMD) and no other documentation of atrophy on chart review and to evaluate possible systemic risk factors for the development of underlying GA lesions.

## Methods

This retrospective study was conducted after receiving approval from the Cleveland Clinic Institutional Review Board. All study-related procedures followed good clinical practice (International Conference on Harmonisation of Technical Requirements for Pharmaceuticals for Human Use [ICH] E6), followed applicable FDA regulations, and adhered to the US Health Insurance Portability and Accountability Act of 1996 and the Declaration of Helsinki.

The primary aim of this study was to identify the prevalence of GA lesions in individuals with an ICD-10 code diagnosis of iAMD. A subset of all patients seen with an ICD-10 code diagnosis for iAMD in at least 1 eye (subcodes H35.3112, H35.3122, H35.3132, and H35.3192) between 2015 and 2022 was screened for this study.

Eyes with a history of a concomitant retinal pathology, such as diabetic retinopathy, retinal vascular occlusion, macular hole, retinal detachment, and uveitis, were excluded. For the final analysis, additional exclusion criteria included eyes that were incorrectly coded as having AMD and eyes in which GA or atrophic changes were noted in a provider’s clinical documentation, including examination, assessment and plan, and imaging interpretation. The index date for each eye was considered the date of the first ICD-10 code diagnosis of iAMD in the patient’s medical record. The patients were seen by optometrists and ophthalmologists of any subspecialty practice at the Cole Eye Institute.

**Figure 1.**
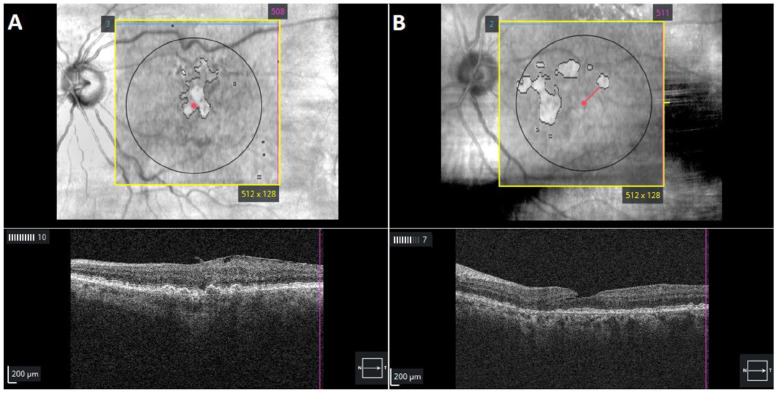
Optical coherence tomography of automated subretinal pigment epithelium illumination showing outlines of (A) a fovea-involving lesion with geographic atrophy (GA) and (B) an extrafoveal GA lesion in patients diagnosed with intermediate age-related macular degeneration.

Eyes meeting the inclusion criteria were analyzed using Advanced RPE Analysis software (Zeiss) (Figure 1) to detect the prevalence of undiagnosed GA lesions on the index date. The central subfield thickness (CST), the sub-RPE illumination area within 5.0 mm of the fovea, and the distance of the nearest lesion edge from the fovea (foveal distance) were collected for each eye. As in previous studies, a sub-RPE illumination value of 0.4 mm^2^ or greater was used as the threshold to identify atrophic lesions.^16^ Eyes flagged as having a sub-RPE illumination value of 0.4 mm^2^ or greater were reviewed by 2 graders (A.S., C.M.) to determine the stage of atrophy, if any, that was present. Staging was according to the Classification of Atrophy Meetings criteria and included incomplete outer retinal atrophy (iORA), complete outer retinal atrophy (cORA), incomplete retinal RPE and outer retinal atrophy (iRORA), and complete RPE and outer retinal atrophy (cRORA).^17^ There was 76% agreement between the 2 graders; conflicting grades were resolved through consensus. If lesions were inappropriately shaded by the algorithm, reshading was performed. Only eyes with iRORA and cRORA were considered to have a GA lesion.

Clinical data were also collected, including age on the index date, race, sex, visual acuity (VA), smoking history, and medical history, including body mass index, hypertension, hyperlipidemia, chronic kidney disease, diabetes, and coronary artery disease.

The prevalence of undiagnosed GA lesions was calculated by dividing the number of eyes identified by graders as having a GA lesion by the total number of eyes meeting the inclusion criteria. Logistic regression analysis was used to identify factors increasing the odds of an undiagnosed GA lesion being present. Eyes with GA lesions were compared with an age-matched and sex-matched group of eyes without lesions; 1:1 propensity score matching used the nearest neighbor method to establish the matched control group. Numerical variables are presented as the mean ± SD, and categorical variables are presented as percentages. Analysis was performed with Excel software (Microsoft Corp) and R software (version 4.2.3, R Project for Statistical Computing).

## Results

### Demographics

**Figure 2.**
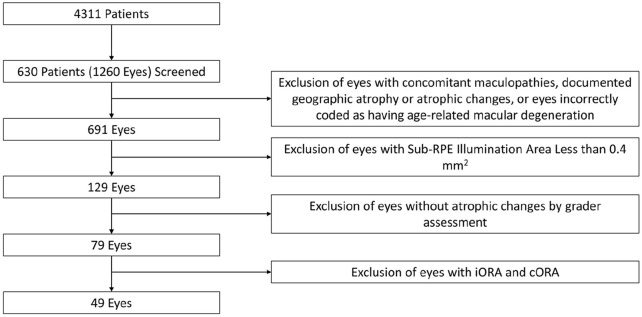
Preferred Reporting Items for Systematic reviews and Meta-Analyses criteria for patient inclusion. Abbreviations: cORA, complete outer retinal atrophy; iORA, incomplete outer retinal atrophy; RPE, retinal pigment epithelium.

The study assessed 1260 of 630 patients for study inclusion. For the final analysis, 91 eyes (7.2% of initial eyes screened for inclusion) were excluded. In total, 690 eyes of 428 patients remained after exclusion (Figure 2). The mean age of the included patients was 79.4 ± 8.4 years. Men comprised 33.6% of the patients, and 94.4% were White. Patients with GA lesions were older on average than patients without lesions (83.4 ± 7.7 years vs 78.9 ± 8.4 years; *P =* .001). The mean best-corrected VA (BCVA) across the sample was 72.4 ± 13.0 letters (Snellen equivalent, 20/35) and was significantly better in patients without GA lesions (*P =* .001). Patients with lesions were more likely than those without lesions to be pseudophakic (70.2% vs 55.0%). Table 1A shows a detailed analysis eye demographics and Table 1B, of patient demographics.

**Table 1A.** Demographics of Eyes With iAMD With or Without GA Lesions.

Variable	All Eyes (N = 690)	GA Lesions (n = 49)	No GA Lesions (n = 641)	*P* Value
Mean BCVA (ETDRS letters) ± SD	72.4 ± 13.0	66.4 ± 13.8	72.9 ± 12.9	.001
Mean IOP (mm Hg) ± SD	15.1 ± 3.3	14.0 ± 3.0	15.2 ± 3.3	.02
Pseudophakic (%)	56.10	70.2	55.0	.04

Abbreviations: BCVA, best-corrected visual acuity; ETDRS, Early Treatment Diabetic Retinopathy Study; GA, geographic atrophy; iAMD, intermediate age-related macular degeneration; IOP, intraocular pressure.

**Table 1B.** Demographics of Patients With iAMD With or Without GA Lesions.

Variable	All Patients (N = 428)	GA Lesions (n = 41)	No GA Lesions (n = 387)	*P* Value
Mean age (y) ± SD	79.4 ± 8.4	83.4 ± 7.7	78.9 ± 8.4	.001
Mean BMI ± SD	28.0 ± 6.0	27.3 ± 6.8	28.0 ± 5.9	.48
Male sex (%)	33.6	41.5	32.8	.27
White race (%)	94.4	95.1	94.3	1.00
Current or former smoker (%)	52.8	63.4	50.0	.19
Chronic kidney disease (%)	15.9	14.6	16.0	.82
Coronary artery disease (%)	20.8	19.5	20.9	.83
Diabetes (%)	22.0	14.6	22.7	.23
Dyslipidemia (%)	69.6	75.6	69.0	.38
Hypertension (%)	65.4	78.0	64.1	.07

Abbreviations: BMI, body mass index; GA, geographic atrophy; iAMD, intermediate age-related macular degeneration.

### Prevalence of GA Lesions

**Figure 3.**
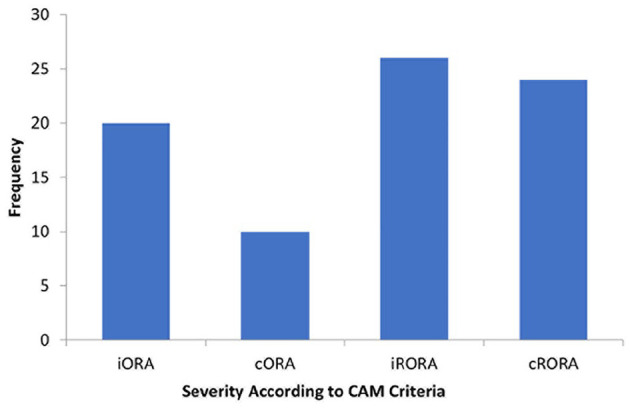
Distribution of atrophy severity. Abbreviations: CAM, Classification of Atrophy Meetings; cORA, complete outer retinal atrophy; cRORA, complete retinal pigment epithelium and outer retinal atrophy; iORA, incomplete outer retinal atrophy; iRORA, incomplete retinal pigment epithelium and outer retinal atrophy.

Of the 690 eyes included in the study, 129 (18.7%) were identified by the automated analysis as having a sub-RPE illumination value of 0.4 mm^2^ or greater. After grading, 79 eyes (11.4%) were found to have some degree of atrophic change. The distribution of atrophy severity among patients according to the Classification of Atrophy Meetings criteria was as follows: 20 eyes (25.3%) had iORA, 10 (12.7%) had cORA, 26 (32.9%) had iRORA, and 23 (28.8%) had cRORA (Figure 3). Therefore, 49 eyes of 41 patients were considered to have a GA lesion, for an overall prevalence of 7.1%.

### Lesion Characteristics

**Figure 4.**
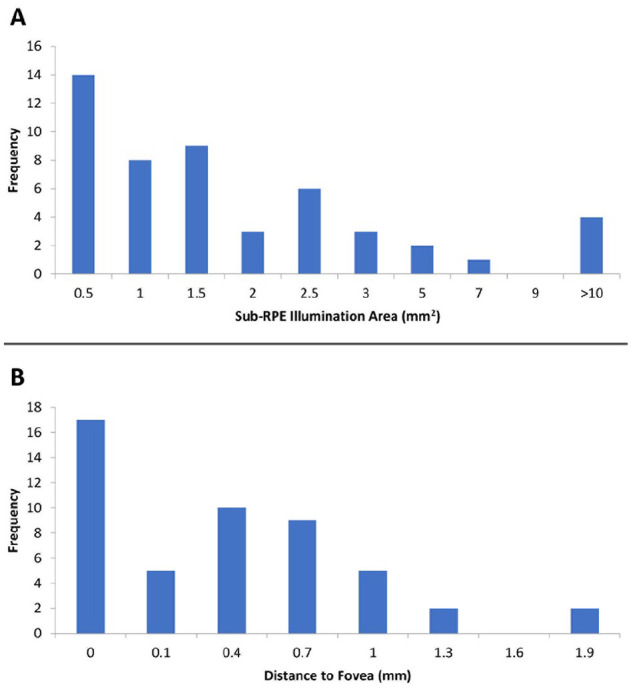
Distribution of (A) lesion sizes and (B) foveal distances. Abbreviation: RPE, retinal pigment epithelium.

Forty-one (83.7%) of 49 images were reshaded. The mean lesion area after reshading of lesions identified through OCT analysis was 2.4 ± 4.0 mm^2^ while the mean foveal distance was 0.4 ± 0.5 mm (Table 2). Figure 4A shows the distribution of GA lesion sizes, and Figure 4B shows the foveal distances across the sample of eyes with GA lesions. The overall mean CST in eyes with GA lesions was 243 ± 32 µm (Table 2). Thirty-four percent of eyes with GA lesions exhibited foveal involvement, defined as a foveal distance of 0 mm. Compared with nonfoveal lesions, the median foveal lesion size was larger (1.7 mm^2^ vs 1.0 mm^2^; *P* = .04); however, there was no difference in CST (Table 3). In addition, although the median BCVA was better in patients with nonfoveal lesions than in those with foveal lesions (69.9 letters [Snellen equivalent, 20/40] vs 63.1 letters [Snellen equivalent, 20/55]), the difference was not statistically significant (*P =* .34).

**Table 2.** OCT Parameters in Patients With GA Lesions.

Parameter	Mean ± SD	Median (IQR)
Sub-RPE illumination area	2.4 ± 4.0	1.2 (1.7)
Foveal distance	0.4 ± 0.5	0.2 (0.6)
Central subfield thickness	243 ± 32	239 (44)

Abbreviations: GA, geographic atrophy; OCT, optical coherence tomography; RPE, retinal pigment epithelium.

**Table 3.** Comparison of Nonfoveal and Foveal and Lesion Characteristics.

Variable	Median (Interquartile Range)		*P* Value
	Nonfoveal Lesions (n = 33)	Foveal Lesions (n = 17)	
Sub-RPE illumination area	1 (1.5)	1.7 (1.9)	.04^a^
Foveal distance	0.5 (0.6)	0	.00
Central subfield thickness	245 (49)	237 (25)	.53
BCVA	69.9 (16.9)	63.1 (10.4)	.34

Abbreviations: BCVA, best-corrected visual acuity; RPE, retinal pigment epithelium.

a Statistically significant.

### Factors Increasing the Odds of GA Lesions

Four eyes in the GA lesion group were missing at least 1 factor of interest in the regression analysis, leaving 45 eyes. Forty-five age-matched and sex-matched control eyes with iAMD and no GA lesions were identified. Table 4 shows the results of the logistic regression analysis used to identify risk factors for GA lesions. None of the factors was found to significantly affect the odds of a patient diagnosed with iAMD having a GA lesion.

**Table 4.** Results of Logistic Regression Comparing iAMD Patients With and Without GA Lesions.^a^

Factor	Odds Ratio	*P* Value
Intercept	0.67	.73
Non-White race	1.00	1.00
Body mass index	1.01	.79
Current/former smoker	1.06	.91
Chronic kidney disease	0.38	.11
Coronary artery disease	0.62	.38
Diabetes	0.55	.33
Hyperlipidemia	0.93	.91
Hypertension	2.14	.20

Abbreviations: GA, geographic atrophy; iAMD, intermediate age-related macular degeneration.

a Only complete cases were used, leaving 45 patients with iAMD and GA lesions and 45 patients without GA lesions.

## Conclusions

The current treatment for GA slows disease progression but does not reverse vision loss. It is therefore imperative to detect early signs of this condition and follow patients over time to assess the risk for progression. Atrophic lesions were identified in 7.1% of eyes in patients with an ICD-10 code diagnosis of iAMD, none of which were documented by clinicians.

OCT, one of many tools available to ophthalmologists, was used in this study to identify lesions. Fluorescein angiography (FA), fundus photography, and dilated fundus examinations can also assist in the diagnosis of atrophic lesions. Including earlier classifications of atrophy in AMD, such as iORA and cORA, increased the rate of detection to 11.4%. These numbers highlight that early signs of GA in this at-risk population with iAMD can be missed if not using the appropriate modalities.

Non-neovascular AMD is a condition that progresses through different stages. The Classification of Atrophy Meetings program established OCT as the standard in the diagnosis and staging of these atrophic lesions.^17^ OCT can identify early stages of the atrophic process, before lesions can be detected on color fundus photographs or FAF.^17^ The final stage of atrophic lesions is cRORA, which is preceded by iRORA, both of which can be assessed consistently with high interrater reliability using the Classification of Atrophy Meetings criteria.^18^ Differentiating between the 2 entities requires several OCT criteria as follows: RPE loss and choroidal hypertransmission of more than 250 μm with signs of overlaying photoreceptor degeneration, such as outer nuclear layer thinning, external limiting membrane loss, and ellipsoid zone or interdigitation zone loss.^17^ In our assessment of the sub-RPE slabs in this study, we were able to accurately detect cRORA and iRORA on the en face images but not the earlier precursors, iORA and cORA. For this reason, only cRORA and iRORA were included in our assessment of atrophic lesions.

Most atrophic lesions in AMD progress over time. In a natural history study, 93.1% of iRORA lesions converted to cRORA lesions within 24 months (median, 14 months), with intraretinal hyperreflective foci and an extrafoveal location of iRORA at baseline associated with a faster rate of progression.^19^ For patients with established GA, there is a variability in lesion growth rates in the literature, ranging from 0.27 to 0.4 mm/year.^20^ Several factors are associated with faster progression, including lesion size,^20^ multifocality (0.19 mm/year vs 0.13 mm/year; 46% higher),^21^ and extrafoveal location (0.22 mm/year vs 0.13 mm/year; 62% higher).^22^ The age of a lesion also affects growth rate, with newer cRORA lesions growing slower than older cRORA lesions. Growth rates of newly diagnosed cRORA lesions (<6 months) were approximately 0.16 mm/year vs 0.26 mm/year for older lesions (>2 years), with square root transformation used to account for the difference in lesion size.^23^ In our cohort, the lesions identified were small (~1 mm^2^), which favored slower growth, and extrafoveal, which had less of an impact on VA at the time of diagnosis. Future studies are needed to assess the growth of these lesions over time and identify which patients would benefit most from treatment and when the best time is to initiate treatment.

Although our study relied on OCT imaging and specifically sub-RPE illumination area analysis, this technology is not without drawbacks. Of 129 eyes, there were 50 instances of false positives with a sub-RPE illumination area greater than 0.4 mm^2^ (38.8%), where the sub-RPE illumination analysis detected atrophy that was deemed by the graders not to be atrophy. Anecdotally, this was often the result of choroidal atrophy, high myopia, subtle epiretinal membranes, and the presence of vitelliform lesions. Thus, although OCT can be a useful tool, it does not replace image analysis by a physician.

Previous imaging studies comparing en face OCT with other imaging modalities, including FAF and FA, have shown that these modalities have good agreement among one another for baseline assessment of lesion size,^24^ with the highest agreement between en face OCT and FAF (*r* = 0.98) after manual correction of SD-OCT segmentation errors.^14^ However, these modalities were assessed in the context of the measurement of a large GA lesion and not as a clinical tool for lesions in the population with iAMD. This study used one of a variety of tools for detecting and measuring GA lesions to estimate the prevalence of early, undiagnosed lesions. However, future head-to-head studies comparing the sensitivity and specificity of various tools for GA lesion characterization would be beneficial in creating screening guidelines for this at-risk population of patients with iAMD.

It has been previously shown that GA ICD-10 coding is undercoded by clinicians. In a low-vision referral center, 24% of patients with GA were coded as having iAMD.^25^ Undercoding and underreporting of GA in patients with concurrent neovascular AMD (nAMD) is also common, with documentation of GA in clinical notes occurring in only 68% of patients with nAMD with confirmed GA on OCT and only 8% given an ICD-10 code for GA in the same cohort.^26^ Lack of GA ICD-10 coding can occur for multiple reasons, including under-recognition of the pathology, lack of easy access to precise diagnostic codes in the electronic medical record, and insurance claims not including all secondary, yet pertinent, diagnoses.^27^

In our cohort, ICD-10 code diagnoses were given by a variety of eye providers, including optometrists, general ophthalmologists, and all ophthalmology subspecialists. Patients were also assessed during a time when there was no treatment for GA, and clinicians may not have grasped the importance of documenting smaller areas of atrophy for patients with iAMD. These findings raise the importance of increased training for eye providers on OCT findings in patients with iAMD. It remains to be seen whether the diagnosis and detection of GA will improve now that treatments exist and clinicians may be more actively looking for them.

In addition to pegcetacoplan, several other therapies are under investigation for the treatment of GA.^28^ Avacincaptad pegol is a pegylated RNA aptamer that binds specifically to complement C5 and is also administered via IVT injection. This medication has met its primary endpoint in phase 2 and phase 3 trials; the mean GA growth (square root transformation) over 18 months was reduced by 30% with a favorable safety profile.^29^ Ocular gene therapy trials (EXPLORE^30^ and HORIZON^31^) have evaluated treatments to restore complement system homeostasis by increasing complement factor 1 protein production with a subretinal administration of an adeno-associated virus 2–based gene therapy delivery system. OpRegen, a human embryonic stem cell–derived RPE cell therapy, is also being investigated in a current phase 1/2a study.^32^

Because of the retrospective nature of this study, it was not possible to determine whether the diagnosis of iAMD was made as a result of a lack of awareness of the atrophic lesion or other factors, a including lack of time or the perception that it would not change clinical management. Given that GA is underreported in ICD-10 coding,^25^ we addressed this by ensuring that no mention of atrophy was listed in the clinical documentation or OCT interpretation. Because this was a cross-sectional study, patients were not followed to determine whether these lesions did indeed progress. Therefore, we are unable to give recommendations with respect to the frequency of screening for early atrophic lesions in patients with iAMD. Given the relatively low number of eyes with GA lesions, our propensity-matched logistic regression was underpowered to detect smaller effect sizes. Also, although the OCT software can detect hypertransmission, the exact borders of the lesions in this cohort required reshading in most cases (83.7%).

Using OCT imaging analysis, atrophic lesions were found in 7.1% of eyes with an ICD-10 code diagnosis of iAMD and no mention of atrophy in the clinical notes. This highlights how OCT can assist in the detection of early GA lesions in patients with iAMD, particularly as GA-specific therapies become more widely available. Future studies should include head-to-head comparisons of various imaging modalities in patients with iAMD as well as longitudinal follow-up of these patients to determine optimal screening guidelines.
